# APOA2: New Target for Molecular Hydrogen Therapy in Sepsis-Related Lung Injury Based on Proteomic and Genomic Analysis

**DOI:** 10.3390/ijms241411325

**Published:** 2023-07-11

**Authors:** Yuanlin Wang, Yan Fan, Yi Jiang, Enquan Wang, Yu Song, Hongguang Chen, Feier Xu, Keliang Xie, Yonghao Yu

**Affiliations:** 1 Department of Anesthesiology, Tianjin Medical University General Hospital, Tianjin 300052, China; wyl1996@tmu.edu.cn (Y.W.); jiangyi@tmu.edu.cn (Y.J.); enquanwang20162021@gmail.com (E.W.); 15822686071@163.com (Y.S.); hongguangchen@tmu.edu.cn (H.C.); sophiaxufeier@gmail.com (F.X.); 2 Department of Critical Care Medicine, Tianjin Medical University General Hospital, Tianjin 300052, China; fanyan1120@tmu.edu.cn

**Keywords:** molecular hydrogen, septic lung injury, proteomics, genome, Mendelian randomization, GWAS, PWAS

## Abstract

Target biomarkers for H_2_ at both the protein and genome levels are still unclear. In this study, quantitative proteomics acquired from a mouse model were first analyzed. At the same time, functional pathway analysis helped identify functional pathways at the protein level. Then, bioinformatics on mRNA sequencing data were conducted between sepsis and normal mouse models. Differential expressional genes with the closest relationship to disease status and development were identified through module correlation analysis. Then, common biomarkers in proteomics and transcriptomics were extracted as target biomarkers. Through analyzing expression quantitative trait locus (eQTL) and genome-wide association studies (GWAS), colocalization analysis on Apoa2 and sepsis phenotype was conducted by summary-data-based Mendelian randomization (SMR). Then, two-sample and drug-target, syndrome Mendelian randomization (MR) analyses were all conducted using the Twosample R package. For protein level, protein quantitative trait loci (pQTLs) of the target biomarker were also included in MR. Animal experiments helped validate these results. As a result, Apoa2 protein or mRNA was identified as a target biomarker for H_2_ with a protective, causal relationship with sepsis. HDL and type 2 diabetes were proven to possess causal relationships with sepsis. The agitation and inhibition of Apoa2 were indicated to influence sepsis and related syndromes. In conclusion, we first proposed Apoa2 as a target for H_2_ treatment.

## 1. Introduction

According to the latest definition, sepsis is identified as a type of life-threatening condition specifically accompanied by an out-of-control inflammatory response [1]. Recent research on sepsis found that globally, there are 50 million cases of sepsis every year, and one-third of patients suffering sepsis in critical care units are not capable of surviving more than 30 days [2,3,4]. The critical care medicine field is striving to take proactive measures in the early diagnosis of sepsis with effective biomarkers for identification and appropriate treatment, especially with specific drugs. At present, two primary treatment means, antimicrobial and supporting, play essential roles in the clinical setting. In addition, a variety of novel treatments have been developed in recent decades, such as monoclonal antibody targeting [5] and immunoglobulin infusion. These novel treatments reduced the mortality of sepsis [6] and protect organs from the inflammatory response by neutralizing toxins and inhibiting toxicity-related factors [7]. It is regrettable that no effective, specific drugs are available yet. Thus, it is an urgent challenge for further research to seek new, effective and non-toxic methods for the treatment of side effects.

Dole et al. first reported the medical effects of hydrogen and indicated that hydrogen had inhibitory effects on tumor growth in mice [8]. Based on current research, 75% of studies on hydrogen therapy have shown significant value in the treatment of multiple diseases, and clinical studies will also be expanded to a wider range of research areas in the future [9]. A previous study found that reduction of inflammation and apoptosis were closely related to the alleviation of lipopolysaccharide (LPS)-induced acute lung injury by molecular hydrogen [10,11]. In addition, hydrogen has also been proven to reverse cardiac dysfunction by down-regulating the expression of TLR4-mediated pro-inflammatory cytokines [12]. Meanwhile, positive effects of hydrogen in the nervous system [13], metabolism [14] and digestive system [15] have also been reported. A randomized double-blind placebo-controlled trial of hydrogen inhalation for Parkinson’s disease (PD) showed that hydrogen inhalation was safe in patients with PD [16]. In a recent randomized controlled trial, 6 months of intake of hydrogen-rich water (HRW) delayed aging by increasing average telomere length and improving DNA methylation [17]. A retrospective study suggested that hydrogen was able to alleviate clinically severe symptoms caused by COVID-19 [18].

The molecular mechanism of hydrogen therapy is complex, including anti-inflammatory and antioxidant effects and the regulation of autophagy and mitochondrial function. In an RNA sequencing study of septic mice, hydrogen deactivated early inflammatory signaling pathways, such as the STAT3 signaling pathway [19]. Meanwhile, hydrogen therapy reversed the overexpression of upstream regulatory genes and multiple cytokine receptor genes in sepsis [19]. In LPS-induced systemic inflammation (SI), 2% molecular hydrogen inhalation prevented fever and hypotension by reducing the expression level of proinflammatory cytokines (TNF-α and IL-1β) and prostaglandin E2 (PGE2) and increasing the level of anti-inflammatory cytokines (IL-10) [20].

With respect to its anti-inflammatory effects, the antioxidant effects of hydrogen are widely accepted. Genes in the antioxidant stress pathway, including *Gpx1*, *Mmp9*, and *Pxdn*, were significantly down-regulated in the hydrogen treatment group (GO: 0006979) [21]. Mechanically, H_2_ can reduce the production of NO by inhibiting the expression of iNOS and eliminating No-derived ONOO^−^ [20]. Additionally, H_2_ could reduce the production of reactive oxygen species (ROS) by mitochondria by inhibiting the production of NADPH oxidase and MDA [22]. Plus, H_2_ can activate Nrf2 and induce the expression of downstream antioxidant factors [23,24]. Hydrogen-rich saline modulates autophagy via the mTOR/TFEB signaling pathway to inhibit LPS-induced acute lung injury and endothelial dysfunction [25]. Notably, hydrogen regulates the mitochondrial dynamics (fusion/division, biosynthesis and autophagy) of sepsis, maintains the normal morphology and function of mitochondria, and plays a role in the prevention and treatment of sepsis [26,27]. Although some omics studies of molecular hydrogen have been conducted [28], protein-level studies only were not enough.

Developments in bioinformatics and multi-omics are providing more possibilities to explore molecular mechanisms and drug targets for sepsis. However, studies in the above fields are still few. The objective of this study was to reveal protein and mRNA targets for molecular hydrogen treatment of sepsis-related acute lung injury. Three parts were included: (i) the combination of quantitative proteomics and transcriptomics helped identify promising target biomarkers; (ii) Mendelian randomization analysis and co-localization analysis of GWAS and PWAS revealed the causal relationships between biomarkers and phenotypes; (iii) experiments on an animal model validated conclusions obtained in the above analysis.

## 2. Results

**Figure 1:**
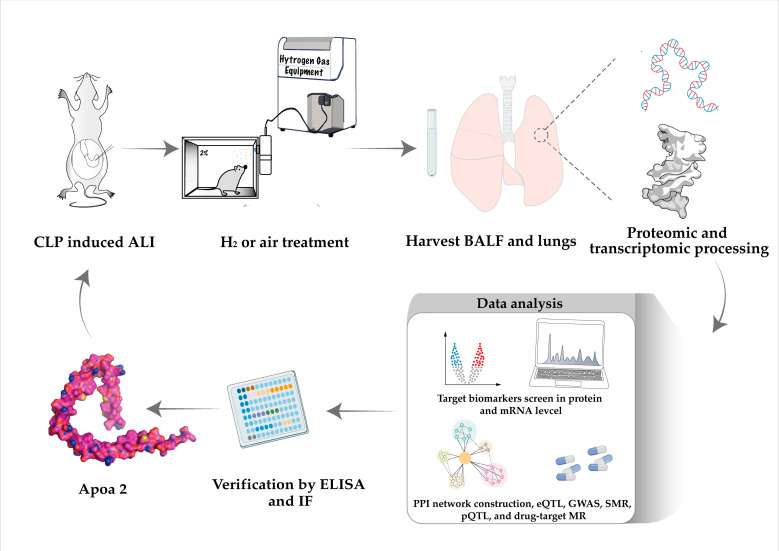
Detailed flow chart of the whole study.

Detailed flow chart of the whole study was shown in Figure 1.

### 2.1. Proteomics Target Proteins Analysis

**Figure 2:**
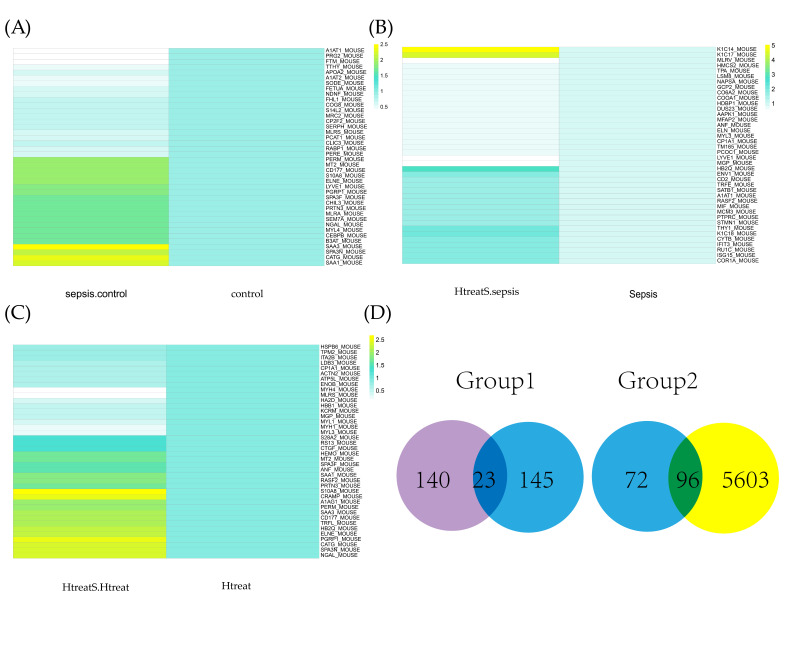
H_2_ gas targeting proteins. (**A**) Top 50 differential proteins sepsis/sham; (**B**) Top 50 differential proteins H_2_ + sepsis/sepsis; (**C**) Top 50 differential proteins H_2_ + sepsis/H_2_ + sham; (**D**) Venn diagram indicating two types of target biomarkers’ identification (group 1 and group 2).

In Figure 2A, differentially expressional peptides are shown; 112 proteins were up-regulated and 56 proteins were down-regulated between sepsis and control. In our analysis, as shown in Figure 2B, 133 up-regulated and 30 down-regulated proteins between H_2_ + sepsis/sepsis were screened. For the sepsis and hydrogen treatment control group, 58 up-regulated and 28 down-regulated proteins were identified (Figure 2C). According to the first screening method, 22 proteins were subsequently identified. According to the second method, 186 proteins were selected. In Venn diagrams, as shown in Figure 2D, the two groups of candidate proteins were exhibited. Further studies were also conducted on these three proteins.

### 2.2. Pathway Analysis and GO Analysis

**Figure 3:**
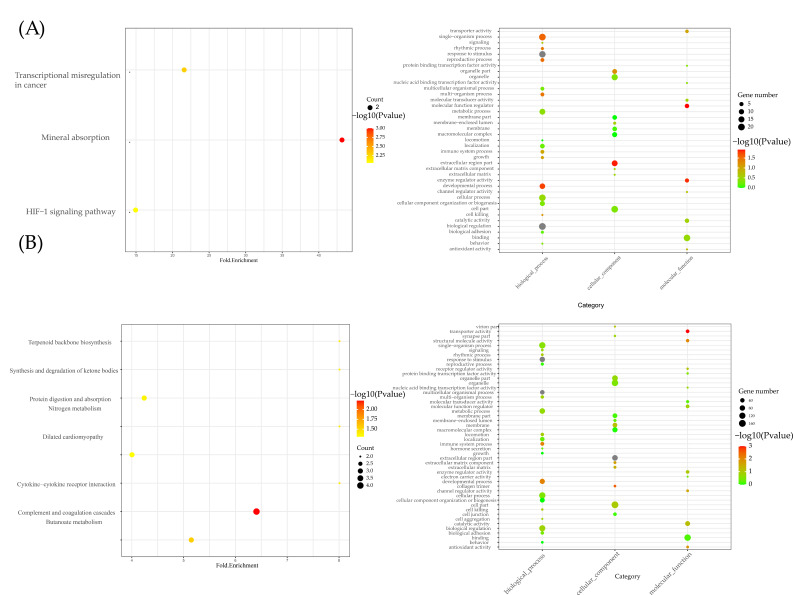
Pathway analysis and GO analysis. (**A**) KEGG and GO results for group 1; (**B**) KEGG and GO results for group 1.

In Figure 3A, three KEGG pathways in group 1 were identified: transcriptional misregulation in cancer, mineral absorption and the HIF-1 signaling pathway. For group 2, as shown in Figure 3B, complement and coagulation cascades and the dilated cardiomyopathy pathway were the two primary pathways. In GO analysis, the extracellular region was enriched in the cell component of the two groups. For biological processes, the response to stimulus and the developmental process were enriched.

### 2.3. Differential Expressional Genes Analysis

**Figure 4:**
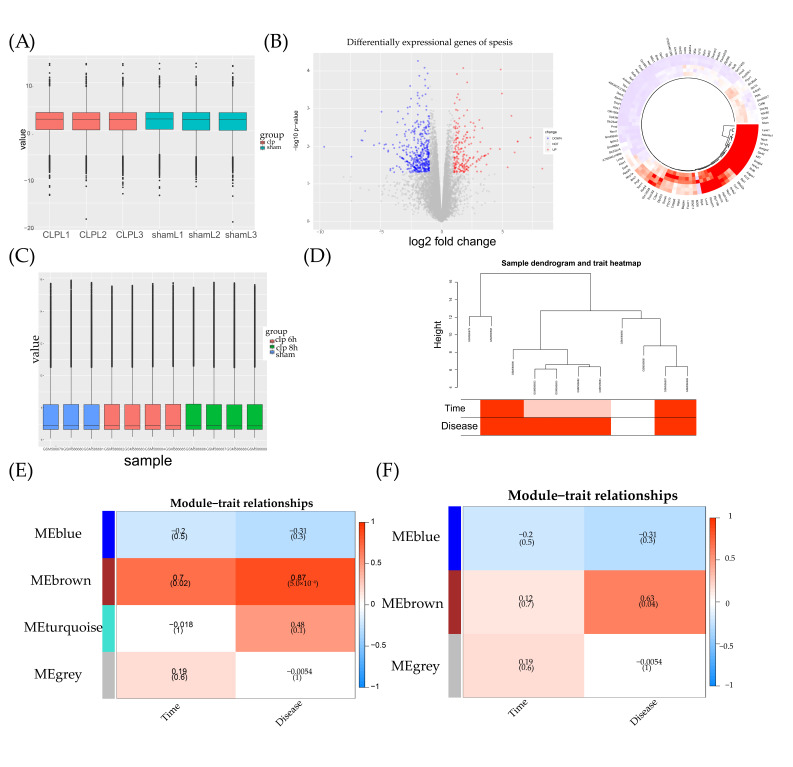
Sepsis-related mRNAs screening. (**A**) Boxplot for mRNA sequencing data acquired from mice model; (**B**) volcano plot and circle heatmap for DEGs; blue indicated down-regulated, while red indicated up-regulated; (**C**) boxplot for GSE23767; (**D**) tree distribution of representative traits and gene expression; (**E**,**F**) module analysis on the basis of dynamic color and merged color distribution.

In Figure 4A, no significant error shifts can be observed in terms of sequencing between the two groups on the histogram. In Figure 4B, all the DEGs are presented in the volcano plot and heatmap. A total of 420 DEGs were up-regulated and 192 DEGs were down-regulated between the sepsis and control group. The top 100 DEGs with the most significant differential expression are also shown in Figure 4B. No significant error shifts can be observed in GSE23767 in Figure 4C. In the WGCNA analysis, the data availability and good clustering results were also verified (Figure 4D). Before merging the assembly modules, four types of dynamic colors were identified (Figure 4E). Three types were identified after merging (Figure 4F). In the following analysis, the brown module had the highest correlation with both disease (0.7, *p*-value = 0.02) and time (0.87, *p*-value = 5 × 10^−4^) phenotypes. After merging, the merged brown module, which included part of the mRNAs in the dynamic brown module, had the highest correlation with disease.

### 2.4. PPI Network Construction

**Figure 5:**
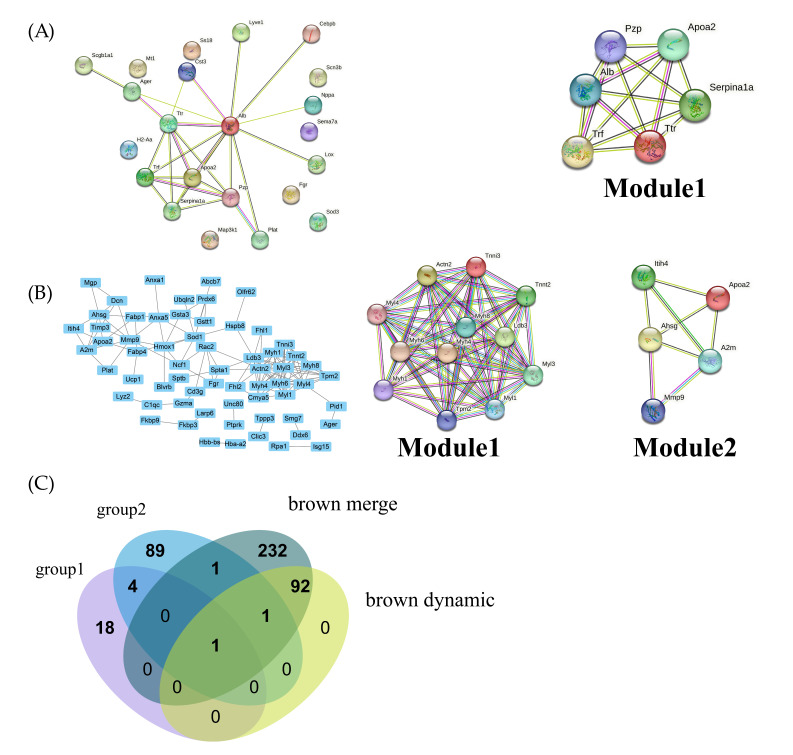
PPI network construction and target biomarker identification. (**A**) PPI network for group 1 proteins and module identification; (**B**) PPI network for group 2 proteins and module identification; (**C**) Venn diagram for target biomarker (protein or mRNA); the four types of color represented four clusters of mRNAs and proteins.

By importing mRNA and protein symbols into the string database, a PPI network was constructed. In our network, disconnected dots were hidden. Module analysis in the PPI network helped to select important modules from the two PPI networks. In our analysis, highly aggregated modules were screened from two PPI networks. In the group 1 module analysis, one module around Apoa2 was screened (Figure 5A). In the group 2 module analysis, two modules were screened (Figure 5B). In both modules, one module was related to Apoa2, suggesting the significance of Apoa2.

### 2.5. Common Hydrogen Treatment Protein Screening

As shown in Figure 5C, Apoa2 was selected from Venn diagrams in our module analysis. Apoa2 was closely related with several pathways: channel regulator activity, developmental process and multicellular organismal process. This result was consistent in both proteomics and transcriptomics.

### 2.6. Results of SMR and Drug-Target MR

**Figure 6:**
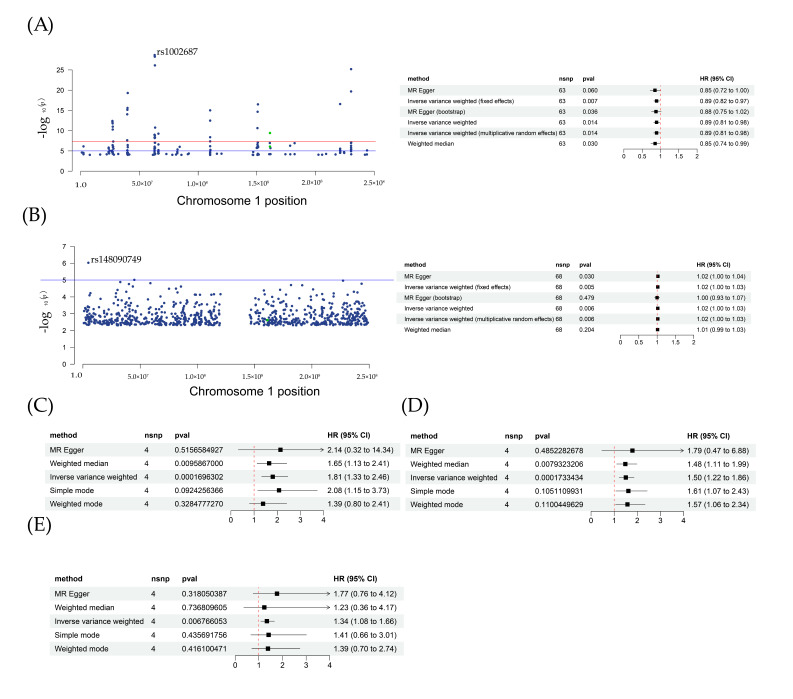
Results of eQTL, pQTL and GWAS analysis. (**A**) Localization of Apoa2 target in HDL and forest plot for HDL and sepsis; green dots represent the SNP used in drug target MR; (**B**) localization of Apoa2 target in type2 diabetes and forest plot for type2 diabetes and sepsis; green dots represent the SNP used in drug target MR; (**C**,**D**), forest plot for Apoa2 mRNA in syndrome target MR, pneumonia and heart failure; (**E**) MR analysis of pQTLs with sepsis.

Firstly, a two-sample MR analysis of HDL and sepsis suggested that HDL played a protective role in sepsis (Figure 6A). In the IVW model, *p*-value = 0.015, OR (95% CI) = 0.89 (0.81–0.98); in the weight median model, *p*-value = 0.03, OR (95% CI) = 0.85 (0.74–0.99). a sensitivity analysis showed that all *p*-values for heterogeneity and the pleiotropy test had no statistical significance. The same results were obtained for MR-PRESSO. In our MR analysis for type 2 diabetes and sepsis, two models, MR Egger and IVW, both exhibited significance (Figure 6B). In MR Egger, *p*-value = 0.03, OR (95% CI) = 1.02 (1.00–1.04). In IVW, *p*-value = 0.006, OR (95% CI) = 1.02 (1.00–1.03). No pleiotropy or heterogeneity was found, as shown in Table 1. These results were also consistent with previous studies showing that type 2 diabetes would induce a decrease in HDL.

In addition, for drug–target MR analysis, two SNPs were selected from the HDL dataset. In a further analysis, as shown in Table 2, the *p*-value for IVW was 0.03, β = −0.77. The protective effects of Apoa2 mRNA activation in sepsis were validated. In the inhibitor experiments, one SNP was selected from the type 2 diabetes phenotype dataset. After MR analysis, showed that β = 0.11, *p*-value = 0.02, Table 2. According to these results, the change in Apoa2 mRNA affected the sepsis outcome. From the eQTL dataset, a total of 225 SNPs of mRNA Apoa2 were screened. SMR analysis results showed that rs3924264 was the top SNP, with the strongest correlation with sepsis traits. In addition, for GWAS analysis, Table 2, OR (95% CI) = 0.91 (0.86–0.98), *p*-value = 0.008; for EQTL analysis, OR (95% CI) = 1.39 (1.34–1.44), *p*-value = 5.27 × 10^−62^; for SMR analysis, OR (95% CI) = 0.55 (0.35–0.86), *p*-value = 0.008. From SMR analysis, a colocalization correlation relationship between Apoa2 mRNA and sepsis was identified.

### 2.7. Syndrome–Target and pQTL MR Analysis

Then, we extracted four SNPs from sepsis datasets to evaluate Apoa2 mRNA effects on two syndromes, pneumonia and heart failure. In Figure 6C, for pneumonia, two models, weight median and IVW were of statistical significance. In weight median, *p*-value = 0.009, OR (95% CI) = 1.65 (1.13–2.41); IVW model, *p*-value = 0.0002, OR (95% CI) = 1.81 (1.33–2.46). In Figure 6D, for heart failure, two models, weight median and IVW were of statistical significance. In weight median, *p*-value = 0.008, OR (95% CI) = 1.48 (1.11–1.99); IVW model, *p*-value = 0.0002, OR (95% CI) = 1.50 (1.22–1.86). In Figure 6E, for pQTLs and sepsis, one model, IVW, was of statistical significance: *p*-value = 0.007, OR (95% CI) = 1.34 (1.08–1.66). The following sensitivity analysis showed that no pleiotropy and heterogeneity occurred. The above results indicated that Apoa2 mRNA had a causal relationship with sepsis-related syndrome, and could induce a bad outcome.

### 2.8. Results of ELISA and IF

**Figure 7:**
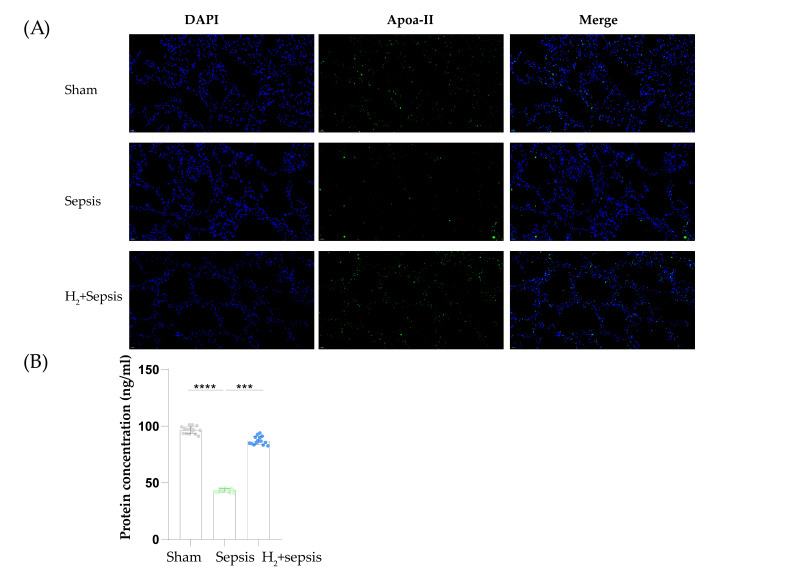
Results of IF and ELISA. (**A**) IF of Apoa2 in lung tissue; green fluorescence represents Apoa2 protein (**B**) ELISA results of Apoa2. *** = *p*-value < 0.001, **** = *p*-value < 0.0001 (*n* = 10).

In immunofluorescence (IF) experiments, Apoa2 level was found to be significantly decreased in sepsis mice lung tissue (Figure 7A). After H_2_ intervention, the level of Apoa2 in the lung was restored. In the next ELISA experiment Figure 7B, Apoa2 protein levels were found to be down-regulated after the CLP model (*p*-value < 0.05, *n* = 10) and up-regulated after H_2_ treatment. H_2_ interventions would reverse negative changes in Apoa2 in the development of sepsis according to our results. These results are also consistent with the proteomics and transcriptomes analysis presented above.

## 3. Discussion

In our analysis, Apoa2 was screened for molecular hydrogen treatment targets. The following experiments also strengthened this conclusion. At the mRNA level, *Apoa2* expression was down-regulated in the sepsis model. Regarding protein levels, by using semi-quantitative and quantitative methods, immunofluorescence and Elisa, we found that the trends at the protein level were as follows: Apoa2 was decreased for sepsis and H_2_ treatment was able to restore the abnormal decrease. Multiple MR analyses also indicated that the Apoa2 gene and protein have a causal relationship with sepsis and related syndromes.

The protective effects of HDL in infectious diseases indicate that a decrease in the small HDL concentration could increase mortality for multiple infectious diseases, including sepsis. The hazard ratio of sepsis risk in the lower blood concentration of HDL (<4.7 µmol/L) will increase to 2.17, 95% CI = 1.37–3.35 [29]. As a complex mixture, HDL is composed of several proteins; Apoa-I and Apoa-II are both found in this particle [30]. Apoa-II, as the second most prominent protein, primarily played stable roles and controlled the size of particles. The increased understanding of hydrogen treatment for sepsis has improved its progress. Most studies, including our previous studies, focused on mitochondrial function disorder and other abnormal-cell-death-related pathways. Mitochondrial mitophagy was found to be affected by hydrogen; the activation of PLINK1 could elicit this beneficial mechanism [31,32]. However, there is research on lipids. In sepsis, the intracellular accumulation of lipid reactive oxygen species (ROS) would induce iron-dependent cell death [33]. Studies involving lipid metabolisms have found that hydrogen-rich saline is capable of reducing lipid peroxidation in lung tissue, which is helpful in protecting normal cells and normal lung functions [34]. The latest theories suggest that immune disorders during sepsis are related to the activation of nuclear factor-κB through upstream receptors such as pattern recognition receptors or toll-like receptors [35]. For multiple pathogenic lipid molecules, toll-like receptors are capable of recognizing these and inducing the subsequent downstream response.

At the Apoa2 protein level, in the PPI network, the PPAR signaling pathway is closely related to Apoa2. A previous study has shown that PPAR-γ would decrease to one quarter compared to normal control levels. Meanwhile, the use of a PPAR-γ agonist could inhibit inflammatory cytokine secretion such as TNF-α. Studies using rosiglitazone to activate PPAR-γ indicated that agonist pre-treatments are capable of decreasing TNF-α serum concentration from 1200 pg/mL to 80 pg/mL [36,37]. Another study focusing on PPAR-α in molecular hydrogen-based sepsis treatment showed that hydrogen could reverse the sepsis-induced reduction in PPAR-α. The expression of PPAR-α was decreased in sepsis-related encephalopathy; 2% H_2_ treatment was able to increase its expression and recover it to normal levels [38]. Along with the combined agonist and hydrogen intervention, the number of apoptotic neurons and the concentration of inflammation cytokines in hippocampi tissues IL-1β and IL-18 both decreased. This showed that the PPAR pathway is a potential target for hydrogen. The Apoa-II mRNA expression was increased through the activation of PPAR-α [39]. Therefore, Apoa2, as a link in the PPAR pathway, also has the potential to be a target of hydrogen.

In existing studies, the effects of *Apoa2* mRNA interventions on sepsis are still unknown. In mice plasma, *Apoa2* and *Apoa1* mRNA both showed a strong relationship with the size and concentration of HDL [40]. In drug target MR analysis, the concentration of HDL was used to identify the activation of *Apoa2*. Four SNPs belonging to *Apoa2* were screened for HDL concentration traits. For type 2 diabetes, the HDL decrease was proved. Therefore, in our study, the change in Apoa-II in type 2 diabetes was seen as an antagonist. Sepsis was seen as a type of severe systemic inflammation. Chronic and acute inflammation were both affected by the expression of *Apoa2*. Serum amyloid A (SAA) is also a component of HDL and could induce localized amyloidosis during the acute phase of inflammation [41]. The metabolism of SAA is highly correlated with the composition of HDL [42]. With the aid of Apoa2^−/−^ mice, one study evaluated its influence on SAA and lung injury. Apoa2^−/−^ could alleviate acute lung injury and decrease the surrounding SAA [43]. For CLP-induced sepsis, Apoa2′s role requires need further study.

In addition, our syndrome Mendelian randomization study revealed an increasing probability of sepsis-related syndrome, pneumonia (OR = 1.65) and heart failure (OR = 1.50) due to the *Apoa2* gene changes in sepsis. When contrasted with the effects of the expression of Apoa-II in HDL, this showed that the negative outcomes of sepsis were positively related to Apoa-II. An upregulated expression of *Apoa2* in cardiomyopathy was reported [44]. Our study indicated the causal relationships between *Apoa2* and heart-related outcomes in sepsis. Some clinical studies also found that an increase in blood plasma HDL-Apoa-II would decrease the mortality caused by heart failure through measuring protein concentration using NMR spectroscopy [45].

A study evaluating the occurrence of acute inflammation after orthopedic surgery showed that small HDL rapidly decreases during acute inflammation [46]. In addition, the pathogenic bacteria of sepsis could produce LPS, which could form a lipid complex like HDL. This type of complex can then competitively bind to HDL and the scavenger receptor B, type I, on the cell membrane surface [47]. This activity is primarily caused by Apoa-II [48]. Therefore, with the excessive increase in Apoa-II, the immune response will speed up and an inflammatory storm could break important organs in a short time. This could also explain why *Apoa2* could affect the progression of sepsis in our analysis. At the same time, our colocalization analysis results showed that mRNA *Apoa2* shared common SNPs with sepsis. Both eQTL analysis and GWAS analysis indicated that these common SNPs have causal effects on the sepsis phenotype. Some complications will induce negative sepsis outcomes. This was consistent with our GWAS study’s conclusion. Our proteomic and transcriptomic analysis primarily focused on the lung. Existing studies on Apoa-II in the lung are few. One study revealed that Apoa-II mRNA was essential to lung development in the embryo [49]. In our analysis, the role of Apoa-II in inflammation and lung function could be validated on the basis of existing research.

There were several limitations to our study. Firstly, our drug target’s experiment was conducted at the genomic level; further proteomic levels were lacking. Secondly, our CLP mice model represented moderate to severe sepsis, while mild sepsis was not the research focus. Thirdly, the causal relationships were constructed on the basis of genomics; further epidemiology for the human population is needed. Future studies will focus on the structural domain and transcriptional-translational regulation of *Apoa2,* influenced by molecular hydrogen, to clarify the mechanisms. Furthermore, the collaborative treatment of molecular hydrogen and HDL through nano-technologies, or other novel ways to increase treatment effects, will also be focused on.

In conclusion, we propose Apoa-II as a promising new target for molecule hydrogen treatment in sepsis.

## 4. Materials and Methods

### 4.1. Animal Model Construction

Firstly, male C57BL/6 (6–8 weeks old, *n* = 20), purchased from the Laboratory Animal Center of the Military Medical Science Academy in Beijing, China, were included in the study. The animals were housed in a suitable environment: room temperature (20–22 °C), a 12 h/12 h light/dark cycle, with an uninterrupted supply of food and water. Then, we randomly grouped these mice into four groups: sham group (*n* = 5), sepsis group (*n* = 5), hydrogen treatment for sepsis group (*n* = 5), and hydrogen treatment for the control group (*n* = 5). The CLP mice model was established according to a previous study [50]. We induced acute lung injury in mice by cecal ligation and puncture (CLP). After inhalation of isoflurane for anesthesia, the mice were placed flat on the operating table. The abdomen was pretreated with hair removal, and the exposed skin of the abdomen was disinfected with iodophor. The abdomen was prepped and disinfected with Iodophor. An incision of approximately 1 cm in length was made along the midline skin, and then the appendix was freed. To avoid the mesenteric artery, approximately 50% of the cecum was ligated with a silk thread. Then, a 21-gauge needle was used to puncture the ligated cecum.

The ligated caecum was punctured twice with a 21-gauge needle. The caecum was returned to the abdominal cavity after a small amount of feces was squeezed out of the intestine. After the incision was closed with a 3–0 surgical suture, saline solution (1 mL) was subcutaneously injected into the mice, and lidocaine cream (Cat# H20063466) was applied to alleviate their pain. The sham group only underwent laparotomy without cecal ligation or perforation. Detailed treatment measures for each group are as follows: mice receiving H_2_ intervention positively inhaled hydrogen for 1 h at the 1 h and 6 h time points after the model was established. After extruding a small amount of feces from the intestine, the cecum was returned to the abdominal cavity. After closing the inner peritoneum and outer skin with 3–0 surgical sutures, saline (1 mL) was injected subcutaneously into the neck of the mice. Then, to relieve pain at the surgical site, lidocaine cream was applied to the surgical area.

### 4.2. Proteomics Collection and Processing

Protein extraction: Lung tissues were collected from four groups of mice, Control, Hydrogen (Htreat), Sepsis, and Treatment (HtreatS), and lysed by RIPA lysis solution containing protease inhibitor mixture. Lung homogenates were centrifuged at 20,000× *g* for 10 min at 4 °C, and the total protein concentration in the supernatant was determined using the 2D Quant kit. Equal amounts of total protein were tested with 12% SDS-PAGE gel for the parallelism of protein extraction and accuracy of quantitative results. Reductive alkylation of proteins was carried out as follows: A final concentration of 5 mM dithiothreitol (DTT) was added to each protein sample and reacted at 30 °C for 30 min. Then, 30 mM iodoacetamide (IAM) was added and incubated for 45 min at room temperature and protected from light. Ten times the volume of −20 °C pre-cooled acetone was added to precipitate the protein and left at −20 °C overnight. The next day, after centrifugation at 20,000× *g* for 10 min at 4 °C, the supernatant was discarded, rinsed by adding 80% acetone, pre-chilled at −20 °C, and left at −20 °C for 1 h. We collected the precipitate and rinsed it twice with pre-chilled acetone. Enzymatic digestion and desalting of proteins were carried out as follows: 300 µL of 0.12 M TEAB were added to protein samples on ice to promote protein precipitation solubilization; then, 6 µg of trypsin was added and digested overnight at 37 °C. A total of 6 µg of trypsin was added again and digested for a second time at 37 °C for 4 h. The reaction was terminated by adding a final concentration of 1% trifluoroacetic acid (TFA). Desalting was carried out with a small C18 SPE column and eluting peptide fractions were drained by a vacuum concentrator. TMT labeling: The peptide was dissolved at a final concentration of 0.5 M TEAB, 50 µg of the peptide was labeled with iTRAQ reagent, and the reaction was carried out at room temperature for 1 h. HPLC pre-separation of labeled peptides was carried out as follows: The labeled peptides, after being re-dissolved with solvent A (10 mM formic acid, pH 10), were separated by conventional high-performance liquid chromatography (HPLC) under alkaline conditions in a reverse gradient. The parameters were set as follows: 0–5 min for 2–10% solvent B (10 mM formic acid, 80% acetonitrile, pH 10), 5–55 min for 10–35%, 55–65 min for 35–90%, 65–70 min for 90%; 70–75 min for 90–92%; 75–80 min for 2% at a flow rate of 1 mL/min. Qualitative and quantitative analyses by liquid mass spectrometry were conducted as follows: Each group was pre-separated by HPLC and dissolved in liquid-phase A (2% ACN, 0.1% FA), centrifuged at 20,000× *g* for 10 min. The supernatant was transferred to a supersampling vial for 1 h for a liquid mass spectrometry analysis of each fraction using a liquid mass spectrometry system with a supersampling volume of about 1 µg.

### 4.3. Transcriptomic Collection and Processing

RNA extraction and quantification were carried out as follows: total RNAs from lung tissues of two groups of mice, Control and Sepsis, were extracted with Trizol reagent. Then, the amount and purity of total RNA were quality-controlled by NanoDrop ND-1000. The integrity of RNA was then examined by Bioanalyzer 2100 and verified by agarose gel electrophoresis. mRNA enrichment and double-stranded cDNA synthesis were carried out: mRNA with PolyA (polyadenylate) was specifically captured by two rounds of purification using oligo(dT) magnetic beads. The captured mRNA was fragmented under 94 °C for 5–7 min. The fragmented RNA was synthesized into cDNA by reverse transcriptase. End repair, addition of A and splicing were carried out as follows: duplex synthesis was performed using E. coli DNA polymerase I with RNase H. These complex duplexes of DNA and RNA were converted into DNA duplexes, while dUTP solution was doped into the duplexes to complement the ends of the duplex DNA to flat ends. An A-base was added to each end to enable ligation to a splice with a T-base at the end. Fragment selection and PCR amplification were carried out as follows: The fragment size was screened and purified using magnetic beads. The second strand was digested with UDG enzyme and then amplified by PCR pre-denaturation.

### 4.4. Data Pre-Processing and Differential Analysis for Proteomics

Firstly, the 21 pre-separated fractions were analyzed individually by liquid mass spectrometry, and 21 raw files of mass spectra were obtained. Then, Proteome Discoverer software (version 2.1) was used to merge into one Mascot input file containing secondary spectrogram information. Mascot software (version 2.6) can also help to perform qualitative and quantitative analysis [51]. The specific steps of the differential analysis for proteins were as follows: (1) count the number of peptides; (2) count the coverage of each protein; (3) count the number of peptides corresponding to each protein. *p*-value < 0.05 was used as the statistical criterion. Then, R package ggplot2 was utilized to exhibit the distribution of differential proteins between the hydrogen treatment sepsis group and sepsis group, sepsis group and control group, hydrogen treatment sepsis group and hydrogen treatment control group. A heatmap was also drawn using the heatmap package [52] to show the special proteins in the whole experiment. In our proteomic analysis, we identified target biomarkers by analyzing the intersection of two protein sets: group1 set (those showing changes in the sepsis versus normal control groups, and those H_2_ treatments under conditions that have induced sepsis can also result in differences in the expression of some proteins), group2 set (those showing changes in the sepsis versus normal control groups, and those without changes in the hydrogen treatment versus normal groups). Venn diagrams were used to screen for biomarkers of these differential expressions. The aim of this criterion was to identify the hydrogen treatment target proteins and mRNAs that were closely related to sepsis.

### 4.5. Data Pre-Processing for Transcriptome

To avoid errors in the subsequent statistical analysis due to the non-normal distribution of the data, log2 transformation was first performed. Then, principal component analysis (PCA) helped to detect the clustering of the mRNA expression matrix of different groups [53]. The limma package [54] was used to detect whether normalization was needed for the matrix. If normalization was needed, the normalization function in the limma package was utilized for corrections.

### 4.6. Differentially Expressional Genes’ Screening

After the above pre-processing procedure, the limma package was used to screen DEGs for hydrogen treatment and sepsis, sepsis and control groups. In our analysis, the criterion for DEGs was set to *p*-value < 0.05, logFC (logFoldchange) ≥ 1 or ≤−1. DEGs with logFC ≥ 1 were named up-regulated DEGs; the others were down-regulated DEGs. Volcano plot and heatmap were used to exhibit DEGs through the ggplot2 R package [55]. Then, we downloaded the GSE23767 dataset from the GEO database. WGCNA [56] algorithms were performed to extract specific mRNAs. The mRNAs included in WGCNA were DEGs screened from transcriptome analysis. The suitable threshold was first identified on the basis of characters. Then, dynamic color and merging color module analyses were conducted to precisely identify the mRNAs with the closest relationship with time and disease. The common mRNAs of the two module analyses were screened for the following analysis.

### 4.7. Protein–Protein Interaction Network and Hub Biomarkers’ Identification

A string database [57] was used to construct the PPI network. By importing target proteins and screening potential proteomics proteins into the string database, a network was constructed. Then, cystoscope software (version 3.8.2) [58] was used to calculate the degree. Important proteins were identified by combining hydrogen treatment target proteins and PPI.

In this study, hub biomarkers were screened by extracting common proteins or mRNAs between proteomic and transcriptomes.

### 4.8. SMR Analysis

Through SMR analysis [59], a tool to test and identify SNPs in the transcripts of mRNAs associated with specific phenotypes, we downloaded mRNA-related transcript information from the eQTL dataset [60]. In this dataset, tissues’ GWAS data from a large population study were included. In SMR analysis, the criterion set for SNPs had a *p*-value < 1 × 10^−5^. Then, phenotype information was downloaded from the ieu-a-4980 GWAS dataset and transformed into a .ma file. SMR software (version 1.3.1) was used to perform this analysis. Three relationship correlations were obtained for a comprehensive evaluation: eQTL, SMR and GWAS.

### 4.9. Two Sample and Drug–Target Mendelian Randomizations for Target mRNA

According to our above proteomics and transcriptomes results, a key mRNA and protein, Apoa2, was screened. Apoa2 was the primary component of high-density lipoprotein (HDL). Type 2 diabetes, which could decrease the expression of Apoa2, was seen as an agonist. Two datasets (met-d-HDL_P and ebi-a-GCST008048) were downloaded from the IEU GWAS website. Data were downloaded from the IEU GWAS database (gwas.mrcieu.ac.uk). In the two-sample MR analysis, the screening criteria for SNPs were as follows: *p*-value < 5 × 10^−8^ or 1 × 10^−5^, r^2^ < 0.001, k = 100,000 kpa. For drug target MR, SNPs with around 100,000 kpa of Apoa2 mRNA were selected. Then, criterion f was: *p*-value < 5 × 10^−3^, r^2^ < 0.3, k = 300 kpa. In drug–target MR analysis, we identified sepsis as an outcome to evaluate the effects of intervening target mRNA. The dataset was obtained from previous GWAS studies on the European population: ieu-b-4980. In addition, to evaluate mRNA’s effects on the sepsis phenotype regarding serious outcomes, we used two complication outcome phenotypes: heart failure and pneumonia. The datasets of these two traits were ebi-a-GCST009541 and ieu-b-4976. Before causal analysis, a sensitivity analysis was performed. Heterogeneity statistics and pleiotropy tests were both used to detect the presence of polyvalence. An R package, MRPRESSO, was also utilized to select IVs with strong polyvalence.

### 4.10. pQTL Analysis for Target Proteins

In this study, we focused on the APOA2 protein; for sentinel and secondary pQTLs, refer to Kari Stefansson [61]. A total of eight pQTLs were obtained. An MR analysis was performed for pQTLs and sepsis phenotype ieu-b-4980.

### 4.11. Enzyme Linked Immunosorbent Assay

Apoa2 levels in the supernatant of bronchoalveolar lavage fluid (BALF) were assayed using ELISA Kits (Suzhou Sizhengbai Biotechnology Co., Ltd., Suzhou, China) according to the manufacturer’s instructions. Mice were anesthetized and the trachea was surgically exposed. The trachea was intubated and lavaged twice with 0.8 mL sterile PBS at room temperature, and BALF was collected. Samples were centrifuged at 500× *g* for 5 min. The remaining supernatant was stored at −80 °C for further analysis.

### 4.12. Immunofluorescence of Lung

The extracted lung tissue was placed in 4% paraformaldehyde for 24 h. Paraffin embedding was then quickly performed. After dewaxing in xylene, dehydration and incubation were conducted, followed by Alexa Fluor 488-conjugated rabbit secondary antibody (Thermo Fisher Scientific, Waltham, MA, USA) for 1 h at room temperature. Then, after the nucleus was labeled with DAPI (Abcam, Cambridge, England), we observed the results using a fluorescence microscope (Olympus, Tokyo, Japan) at 400× magnification.

## 5. Conclusions

Our study proposed that APOA2 has promising protective and curative values in sepsis-related lung injury. Regarding protein and mRNA levels, our conclusions were validated. However, the integrative application of hydrogen and APOA2 was not verified. In addition, the HDL was capable of developing into carriers that could be imported into hydrogen in an in vivo environment. This will be our next research focus.

## Figures and Tables

**Table 1:** Results of sensitivity analysis.

	Cochran Q-Test of IVW	Egger Intercept	MR-PRESSO
HDL and sepsis	Q = 74.7 *p*-value = 0.13	Egger intercept = 0.003 *p*-value = 0.48	Global test = 77.2 *p*-value = 0.16
Type 2 diabetes and sepsis	Q = 68.9 *p*-value = 0.38	Egger intercept = −0.004 *p*-value = 0.51	Global test = 71.5 *p*-value = 0.43
Apoa 2 and pneumonia	Q = 7.04 *p*-value = 0.13	Egger intercept = −0.01 *p*-value = 0.51	Global test = 12.09 *p*-value = 0.33
Apoa 2 and heart failure	Q = 4.39 *p*-value = 0.22	Egger intercept = −0.01 *p*-value = 0.67	Global test = 7.13 *p*-value = 0.45
pQTLs	Q = 1.93 *p*-value = 0.59	Egger intercept = −0.02 *p*-value = 0.58	Global test = 2.73 *p*-value = 0.96

**Table 2:** Results of SMR analysis and drug target.

	β_GWAS	P_GWAS	β_eQTL	P_eQTL	β_SMR	P_SMR
Apoa2	−0.0375	0.008	0.143	5.26 × 10^−62^	−0.263	0.011
Agonists	−0.77	0.03				
Antagonists	0.11	0.02				

## Data Availability

The datasets supporting the conclusions of this article are available in the IEU Open GWAS, deCODE and GEO databases. More original data can be acquired from the corresponding author and Supplemental Materials.

## Supplementary Materials

The following supporting information can be downloaded at: https://www.mdpi.com/article/10.3390/ijms241411325/s1.
